# Cost-Effective and Scalable Clonal Hematopoiesis Assay Provides Insight into Clonal Dynamics

**DOI:** 10.1016/j.jmoldx.2024.03.007

**Published:** 2024-07

**Authors:** Taralynn Mack, Caitlyn Vlasschaert, Kelly von Beck, Alexander J. Silver, J. Brett Heimlich, Hannah Poisner, Henry R. Condon, Jessica Ulloa, Andrew L. Sochacki, Travis P. Spaulding, Ashwin Kishtagari, Cosmin A. Bejan, Yaomin Xu, Michael R. Savona, Angela Jones, Alexander G. Bick

**Affiliations:** ∗ Vanderbilt Genetics Institute, Vanderbilt University School of Medicine, Nashville, Tennessee; † Department of Medicine, Queen's University, Kingston, Ontario, Canada; ‡ Department of Medicine, Vanderbilt University Medical Center, Nashville, Tennessee; § Program in Cancer Biology, Vanderbilt University School of Medicine, Nashville, Tennessee; ¶ Department of Biomedical Informatics, Vanderbilt University Medical Center, Nashville, Tennessee; ‖ Department of Biostatistics, Vanderbilt University Medical Center, Nashville, Tennessee; ∗∗ Vanderbilt Ingram Cancer Center, Vanderbilt University Medical Center, Nashville, Tennessee; †† Center for Immunobiology, Vanderbilt University Medical Center, Nashville, Tennessee; ‡‡ Division of Genetic Medicine, Vanderbilt University Medical Center, Nashville, Tennessee

## Abstract

Clonal hematopoiesis of indeterminate potential (CHIP) is a common age-related phenomenon in which hematopoietic stem cells acquire mutations in a select set of genes commonly mutated in myeloid neoplasia which then expand clonally. Current sequencing assays to detect CHIP mutations are not optimized for the detection of these variants and can be cost-prohibitive when applied to large cohorts or to serial sequencing. In this study, an affordable (approximately US $8 per sample), accurate, and scalable sequencing assay for CHIP is introduced and validated. The efficacy of the assay was demonstrated by identifying CHIP mutations in a cohort of 456 individuals with DNA collected at multiple time points in Vanderbilt University's biobank and quantifying clonal expansion rates over time. A total of 101 individuals with CHIP/clonal cytopenia of undetermined significance were identified, and individual-level clonal expansion rate was calculated using the variant allele fraction at both time points. Differences in clonal expansion rate by driver gene were observed, but there was also significant individual-level heterogeneity, emphasizing the multifactorial nature of clonal expansion. Additionally, mutation co-occurrence and clonal competition between multiple driver mutations were explored.

Although DNA is frequently thought of as fixed at conception, recent studies have described how the genome changes as a natural consequence of aging. Telomeres shorten, mtDNA evolves in copy number and sequence, and the nuclear genome accumulates mutations.^1^, ^2^, ^3^ Hematopoietic stem cells—the self-renewing progenitors of all circulating blood cells—accumulate mutations as they divide. Individual hematopoietic stem cells acquire an estimated 200 mutations per decade genome-wide, with 1 mutation per decade occurring within an exonic region.^4^, ^5^, ^6^ In the vast majority of cases, these somatic mutations confer little to no advantage to the cell, but infrequently fitness-increasing driver mutations confer a proliferative advantage.

Clonal hematopoiesis of indeterminate potential (CHIP) is an age-related condition that is characterized by the acquisition of a somatic driver mutation in a hematopoietic stem cell that leads to an expanded blood cell lineage, termed a *clone*.^7^^,^^8^ The condition increases in prevalence with age, and occurs in >10% of individuals over the age of 70 years.^9^^,^^10^ CHIP confers increased risks for hematologic cancer,^2^ cardiovascular disease,^11^ chronic obstructive pulmonary disease,^12^ liver disease,^13^ kidney disease,^14^^,^^15^ osteoporosis,^16^ and overall mortality.^11^ The minimal variant allele fraction (VAF) used as a threshold to define CHIP is 2% (corresponding to 4% of circulating diploid cells).

CHIP-associated morbidity generally increases proportional to the VAF.^2^^,^^11^^,^^17^^,^^18^ Therefore, the ability to identify individuals who will likely have a faster clonal growth rate, leading to a higher VAF, is important to prevent unfavorable CHIP outcomes. However, quantifying the clonal expansion rate is difficult due to a current scarcity of multi–time point data, precluding observation of these behaviors. In order to understand the factors that impact clonal behavior over time, identifying CHIP accurately and both at a low cost and at scale is essential.

CHIP can be identified in peripheral blood through a variety of sequencing approaches, including whole-genome sequencing,^9^^,^^18^ whole-exome sequencing,^2^ or targeted sequencing.^19^^,^^20^ Whole-genome/exome sequencing is expensive and has limited sequencing depth, making it an unsuitable solution to the large-scale detection of CHIP clones. Targeted amplicon–based approaches are less expensive (approximately US $30 to $50 per sample) but are limited in size, and technical challenges preclude robust detection of specific common CHIP loci, such as *ASXL1* or *SRSF2*, where the high GC content and repetitive sequences make amplicon design difficult. Conversely, hybrid capture–based approaches can overcome some of the shortcomings of amplicon design but historically have been significantly more expensive (US $50 to $150 per sample).

To address this sequencing limitation, a highly cost-effective hybrid capture–based targeted gene panel was developed that encompasses 95% of CHIP mutations found in the general population^9^ by sequencing 22 CHIP genes at high depth of coverage (approximately 2000×) (Figure 1). This method increased CHIP detection accuracy and allowed even small clones to be detected in the blood.

**Figure 1:**
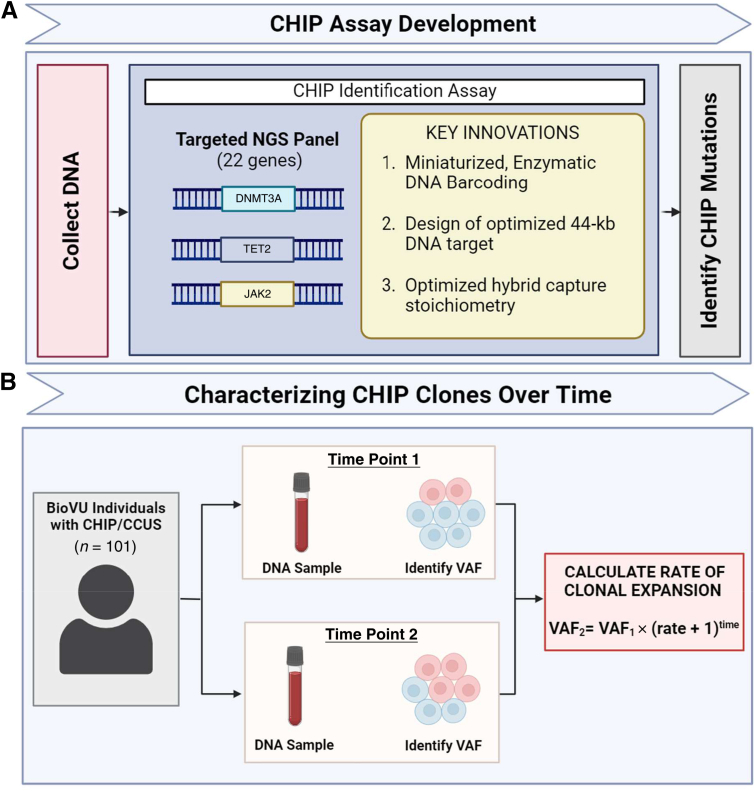
Study design. **A:** Schematic representation of CHIP identification assay. DNA is extracted from the blood and tested for CHIP mutations using the low-cost, scalable targeted assay that tests for 22 specific genes and positions. CHIP mutations are identified and the variant allele fraction (VAF) is estimated. **B:** Schematic representation of characterizing CHIP clones over time in BioVU. The CHIP assay was applied to a cohort using Vanderbilt BioVU with multiple blood samples over time. CHIP/CCUS was identified in one or both time points and VAF was estimated, allowing for the estimation of clonal growth rate over time and characterization of clonal behavior.

As a demonstration of the potential utility of this tool, the assay was applied to a multi–time point blood draw cohort of individuals from the Vanderbilt University's biobank (BioVU, a biorepository of de-identified DNA extracted from discarded blood collected during routine clinical testing; Nashville, TN) (*n* = 456) and identified 101 individuals with CHIP/clonal cytopenia of undetermined significance (CCUS). The clonal expansion rate was quantified on a per-individual basis, which allowed for the identification of the driver genes with faster or slower mean rates of expansion. In individuals with multiple CHIP driver genes, instances of opposing clonal behavior were observed, possibly indicating clonal competition. Together, this method and the subsequent findings will help to better characterize CHIP clonal behavior over time and to identify the specific CHIP mutations at highest risk for rapid expansion.

## Materials and Methods

### Vanderbilt BioVU Samples

All BioVU participants provided informed consent. The Vanderbilt University Medical Center's Institutional Review Board oversees BioVU and approved this project (Institutional Review Board approval 201783). Additional DNA samples were collected as the original DNA sample was depleted, and as a result, approximately 30,000 BioVU participants had blood samples taken at multiple time points over ≤15 years.^21^ The 456 BioVU participants with DNA samples sequenced for this study were selected based on signals of somatic mosaicism from the BioVU Multi-Ethnic Genotyping Array (MEGA; Illumina, San Diego, CA) to maximize the number of individuals with longitudinal CHIP trajectories (Supplemental Figure S1 and Supplemental Table S1). DNA extraction for BioVU leveraged the FlexSTAR instruments (AutoGen, Holliston, MA) and was quantified with NanoDrop and the Quant-iT PicoGreen assay (Thermo Fisher Scientific, Waltham, MA).

While all 456 individuals had samples sequenced on the CHIP assay at multiple time points, the 101 individuals with data used in downstream analyses were a subset of those individuals with at least one CHIP mutation (VAF >2%) and without a blood cancer diagnosis (Supplemental Figure S1). Patient charts were reviewed to determine blood cancer status and to extract other relevant phenotypic and demographic data. Blood count data were not available for these individuals, meaning that cytopenia could not be ruled out, so these individuals were classified as CHIP/CCUS. Of the 101 individuals with detected CHIP mutations, 79% had a mutation with a VAF of >2% at both time points, while the remaining individuals had a mutation with a VAF of >2% at only one time point and a VAF of <2% at the other time point.

### Development of a CHIP Sequencing Assay with a Cost of Less Than US $10 per Sample

A novel, highly scalable and highly cost-effective method was developed to identify individuals with CHIP mutations (Figure 1A and Supplemental Table S2). In order to prioritize which CHIP genes to include in the panel, the distribution of genes in 4229 individuals with CHIP mutations from the Trans-Omics for Precision Medicine (TOPMed) cohort was analyzed. As has been previously described,^9^ CHIP has a skewed distribution: >60% of CHIP mutations are found in just two genes (*DNMT3A* and *TET2*). More than 95% of CHIP mutations are present in the 24 most common genes (Supplemental Figure S2).^9^ Building on this analysis, a specific set of DNA regions that encompass just approximately 44 Kb of DNA but that encompass >95% of all CHIP mutations observed in the general population was identified. The genes included *ASXL1, ASXL2, BRCC3, CBL, DNMT3A, ETNK1, GNAS, GNB1, IDH1, IDH2, JAK2, KIT, KRAS, MPL, NRAS, PPM1D, SETBP1, SF3B1, SRSF2, TET2, TP53,* and *U2AF1* (Supplemental Table S3). The cost of sequencing such a small region of DNA, even at >1000× coverage, is negligible [<$0.30 on a NovaSeq 6000 (Illumina) with an S4 flow cell]. Hybrid capture oligonucleotide probes were designed to selectively amplify this set of genomic intervals (Twist Bioscience, South San Francisco, CA).

To generate a highly scalable and cost-efficient method for selectively sequencing these CHIP DNA intervals, a scalable genomic library preparation method was developed. The method was designed to be compatible with the hybrid capture system to enable efficient processing of genomic DNA from multiple individuals at the same time through DNA barcoding. This approach leverages the Twist Library Preparation Enzymatic Fragmentation Kit version 2.0 (catalog number 104207; Twist Bioscience). Comparison of standard mechanical fragmentation and this enzymatic library preparation demonstrated highly concordant results. The manufacturer's protocol was further optimized through 5× miniaturization of the amount of input reagents for each unit of DNA, which significantly reduced the cost.

The hybrid capture technology was optimized to selectively enrich CHIP regions of interest from the pooled DNA libraries. With the custom-designed hybrid oligonucleotide probes, 12 times the number of hybridization reactions as specified in the manufacturer's protocol could be performed (96 DNA plex samples for each reaction of hybrid capture probes). The entire pipeline was established on a standard liquid handling robot platform (Biomek I7; Beckman Coulter, Brea, CA) to enable preparation and sequencing (on a single liquid handling robot) of up to 5000 samples per week.

Utilizing this approach, the CHIP-enriched DNA libraries were sequenced on standard next-generation sequencing chemistry (Illumina NovaSeq 6000). These libraries were compatible with other standard barcoded libraries such that it was possible to spike in 1% to 5% of the CHIP assay to the total flow cell pool, which typically would contain whole-genome sequencing samples. This technique enables sequencing smaller batches of several hundred samples at a time, rather than waiting for the tens of thousands of samples it would take to fill entire flow cells with just the CHIP assay. At the same time, this approach took advantage of the greater per-base cost efficiency afforded by the larger flow cells.

Putative somatic mutations were identified in the aligned sequencing reads using the Mutect2 version 4.1.0.0–GATK version 4.1.4.1 software package, and filtering was applied to identify variants that met previously described criteria for CHIP.^18^ Variants with total low read depth (<100), low variant allele read depth (<3), and/or a VAF below the threshold for CHIP (<2%) were removed from the data set.

### Calculating the Rate of Clonal Growth

Clonal fitness was calculated on a per-individual basis using an exponential growth rate model:

(1) $${\text{VAF}}_{2}={\text{VAF}}_{1}\text{∗}{\left(\text{CF}+1\right)}^{\text{Time}}$$

Based on this exponential model, clonal fitness was calculated as such:

(2) $$\text{CF}=\left\{{\left({\text{VAF}}_{2}/{\text{VAF}}_{1}\right)}^{\left[1/\text{Time}\right]}\right\}-1$$

In individuals with a CHIP mutation(s) VAF of >2% at only one time point, the missing VAF was set to 0.001 to reflect the sequencing lower detection limit of the assay.

### Classifying Clonal Trajectories

Each CHIP clone was classified as having a stagnant, expansion, or reduction trajectory. To do this, the bottom 10% of clones based on the absolute value of their growth rate were categorized as stagnant. This corresponded to a <2% annual growth rate. In the remaining 90% of clones, those with a positive growth rate were grouped into the expansion category, and clones with a negative growth rate were grouped into the reduction category.

Individuals with two driver mutations were also grouped into sub-clone and distinct clone categories (Supplemental Figure S3). These categories were determined by *z* scoring of the stagnant, expansion, and reduction clone groups separately, producing an individual *z* score for each clone. Individuals with a <0.6 difference in *z* scores between clones growing in the same direction were assigned to the sub-clone category. Individuals with clones growing in opposite directions or whose clones had a ≥0.6 difference in *z* scores were categorized as having distinct clones.

### Association Between Clonal Expansion Rate and Participants' Characteristics

Using a linear/logistic regression model, associations were tested between CHIP expansion rate and age, biological sex, self-reported race, ethnicity, body mass index, and height individually. CHIP driver gene was used as a covariate in each model.

### Statistical Methods

Statistical analyses were performed using R version 4.3.2 (*https://cran.rstudio.com/bin/windows/base/old/4.3.2*). R packages used were ggplot2 (*https://ggplot2.tidyverse.org*), magrittr (*https://cran.r-project.org/web/packages/magrittr/index.html*), dplyr (*https://cran.r-project.org/web/packages/dplyr/index.html*), and data.table (*https://www.rdocumentation.org/packages/data.table/versions/1.15.4*). Regressions were performed in R using either a linear or logistic regression model and the co-occurrence of driver mutations was tested using χ^2^ tests.

## Results

### CHIP Assay Validation

To quantify the lower limit of detection of the method, a limiting dilution experiment was performed in which a DNA sample with known genotype was combined at serial fixed ratios with a second sample of known genotype (Supplemental Figure S4). These ratios were utilized to identify the expected VAF of a given variant. The method robustly detects variants present in >1% of DNA. Furthermore, beneath this 1% threshold, variants were detected down to approximately 0.3% VAF, but with less accuracy for the estimated VAF.

For further validation of the assay in comparison to a Clinical Laboratory Improvement Amendments reference laboratory test gold standard (myeloid next-generation sequencing panel; Vanderbilt Molecular Diagnostics Laboratory, Nashville, TN), a set of clinical patient samples from the Vanderbilt CHIP clinic were considered. All 41 patients with CHIP mutations from the clinical assay were identified as having CHIP mutations on the assay with a high concordance of VAF between the two assays (*R*^2^ = 0.73, *P* = 0.34). As the blood used for clinical next-generation sequencing was derived from a bone marrow biopsy sample, and BioVU samples were peripheral blood typically collected years before the clinical assay, a perfect correlation was not expected.

### CHIP Prevalence and Characteristics in the BioVU Cohort

The CHIP assay was applied to DNA samples from a set of 456 individuals with two or more samples available from different time points in BioVU (Figure 1B). These 456 individuals were selected from a pool of approximately 30,000 individuals with data available from multiple blood draws, which was a subset of approximately 300,000 general BioVU participants (Supplemental Figure S1). These individuals were selected based on a suggestion from the BioVU MEGA intensity data that they were likely to have a CHIP driver mutation(s) in *DNMT3A*, *TET2*, or *JAK2.* These individuals were of increased age (means ± SD: time point 1, 52.5 ± 19 years; time point 2, 57 ± 19 years); 60% were female and 81% were Caucasian (Supplemental Table S1). Samples were sequenced to a mean depth of 1725×. Of these individuals, 173 (34%) had clonal hematopoiesis driver mutations with a VAF of >2% at at least one time point. Data from 72 individuals were removed based on a blood cancer diagnosis that either pre-dated, or occurred within 6 months of, the DNA collection, suggesting that they had blood cancer rather than CHIP.

**Figure 2:**
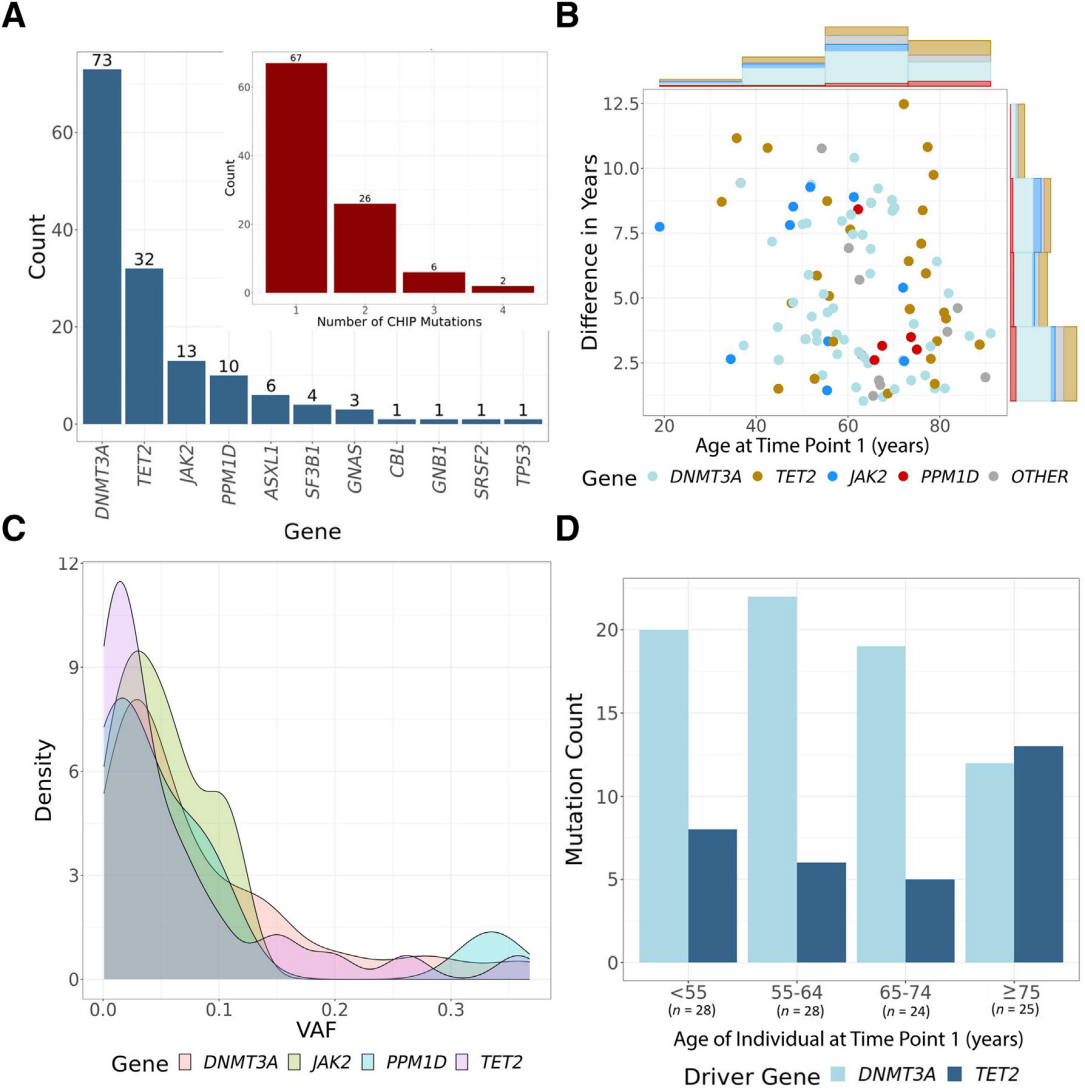
CHIP detected in the BioVU cohort. **A:** Counts of mutations per driver gene in the cohort, and counts of CHIP driver mutations in individuals. *DNMT3A* and *TET2* are the most common driver genes, and the vast majority of individuals have one CHIP driver mutation. **B:** Distribution of age at time point 1, and differences between time points in each individual in the cohort. Histograms along the axes reflect total counts as well as the driver-gene distribution. **C:** Distribution of variant allele fraction (VAF) at time point 1 across the most common driver genes. There were no significant differences between driver genes. **D:** Distribution of *TET2* and *DNMT3A* driver mutations across age categories. The sample sizes refer to the numbers of mutations per age category (not the numbers of individuals).

The final cohort included 101 individuals with CHIP/CCUS and 145 driver mutations (Supplemental Table S4). Of these individuals, 66% had a single CHIP driver mutation, 26% had two mutations, and 8% had three or more mutations (Figure 2A). The most common CHIP driver mutation was in *DNMT3A,* followed by *TET2* (Figure 2A), which is consistent with previously reported driver-gene distributions.^9^ A total of 55% (40/73) of mutations in *DNMT3A* were at recurring hotspots R882C/R882H, likely because these mutations were directly genotyped on MEGA. In a separate analysis, mosaic chromosomal alterations were detected across the BioVU cohort, using previously described methods,^22^^,^^23^ and cross referenced with those from the individuals with CHIP/CCUS. Nine percent (9/101) of individuals also had a mosaic chromosomal alteration, with the majority in the X chromosome. Two of nine individuals had copy-neutral loss of heterozygosity mutations in chromosome 4 in addition to a *TET2* mutation.

In the subset of individuals with CHIP/CCUS, the mean ages at time points 1 and 2 were 65 years (range, 18 to 91 years) and 70 years (range, 26 to 94 years), respectively (Supplemental Table S1). The mean difference between the two time points was 5.4 years (range, 1 to 12.5 years) (Figure 2B). The mean VAFs were 8% and 11.5% at time points 1 and 2. The mean VAFs did not significantly differ between CHIP driver genes (Figure 2C). In individuals aged ≥75 years, *TET2* surpassed *DNMT3A* as the most common CHIP driver mutation (Figure 2D).

### Clonal Expansion Rate Across Driver Genes

**Figure 3:**
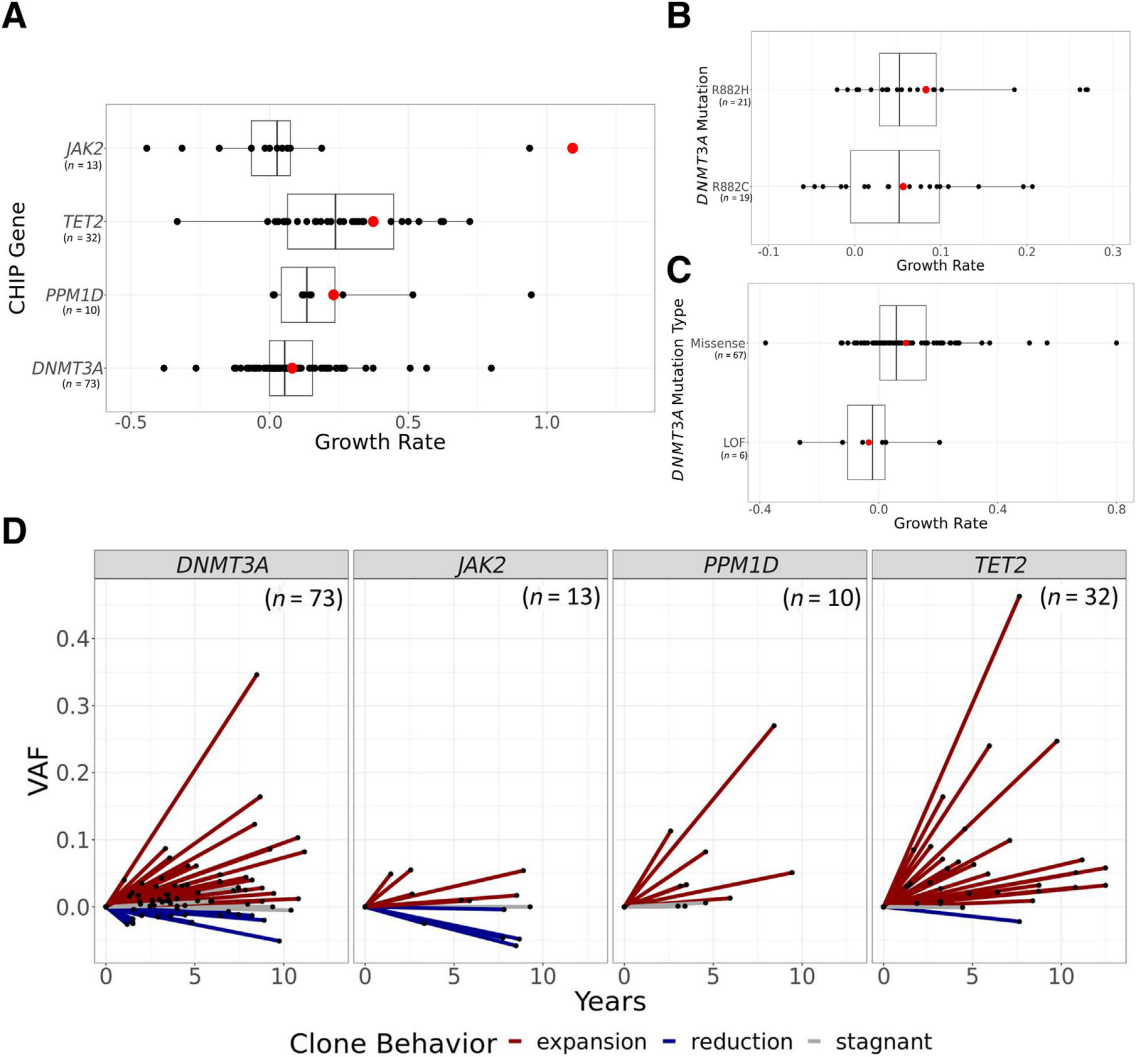
CHIP clonal behavior, by driver gene. **A:** Mean growth rate across the most common driver genes in individuals with CHIP mutations. Each point represents one CHIP mutation. Genes are arranged in descending order on the *y* axis based on mean growth rate, and the *x* axis is limited and excludes values of >1.2. Mean growth rates are marked by dots. **B** and **C:** Mean growth rates (dots) of *DNMT3A* hotspot mutations (**B**) and missense and loss-of-function (LOF) mutations (**C**). **D:** CHIP clone trajectories, shown as change in variant allele fraction (VAF) over time. Each **line** represents one clone, the length of the line represents years between time points, and the slope of the line represents net change in VAF over time. **Lines** are colored by clonal-behavior category and are stratified by CHIP driver gene.

Given that different CHIP driver genes can confer different risks and mechanisms of action,^9^^,^^10^^,^^24^ expansion rates were compared between different driver genes. Across mutations shared by at least 10 individuals, *JAK2* clones showed the fastest rate of expansion on average (109% growth per year) and *DNMT3A* clones showed the slowest rate (8% growth per year) (Figure 3A). To eliminate the possibility of expansion rates being skewed by the presence of multiple clones in an individual, a sensitivity analysis was performed that restricted the individuals to only those with a single CHIP driver mutation. Although this reduced the sample size by 30%, *JAK2* clones remained the fastest growing (178% growth per year), and *DNMT3A* clones remained the slowest growing (5% growth per year).

Of the 73 *DNMT3A* mutations, 21 were at R882H and 19 were at R882C, which are known hotspots. The difference in expansion rates observed between these two hotspot mutations was not significant (*P* = 0.63) (Figure 3B). Stratifying *DNMT3A* mutations into missense versus loss of function identified a nonsignificant trend toward increased growth among individuals with missense mutations (*P* = 0.08) (Figure 3C).

### CHIP Clonal Dynamics

Based on the change in VAF between the time points, each clone was classified as expansion, reduction, or stagnant. A total of 78% of clones expanded over time, suggesting that once an individual acquires a CHIP mutation, that clone tends to keep growing. However, instances of clonal reduction were observed across driver mutations (Figure 3D), even with restriction to individuals with only one driver mutation. The clones that were reduced composed 46% of *JAK2* clones, 40% of *ASXL1* clones, 26% of *DNMT3A* clones, 13% of *PPM1D* clones, and 6% of *TET2* clones. *ASXL1* clones showed the fastest rate of reduction on average (13% reduction per year), and *TET2* clones showed the slowest rate of reduction (0.6% reduction per year).

**Figure 4:**
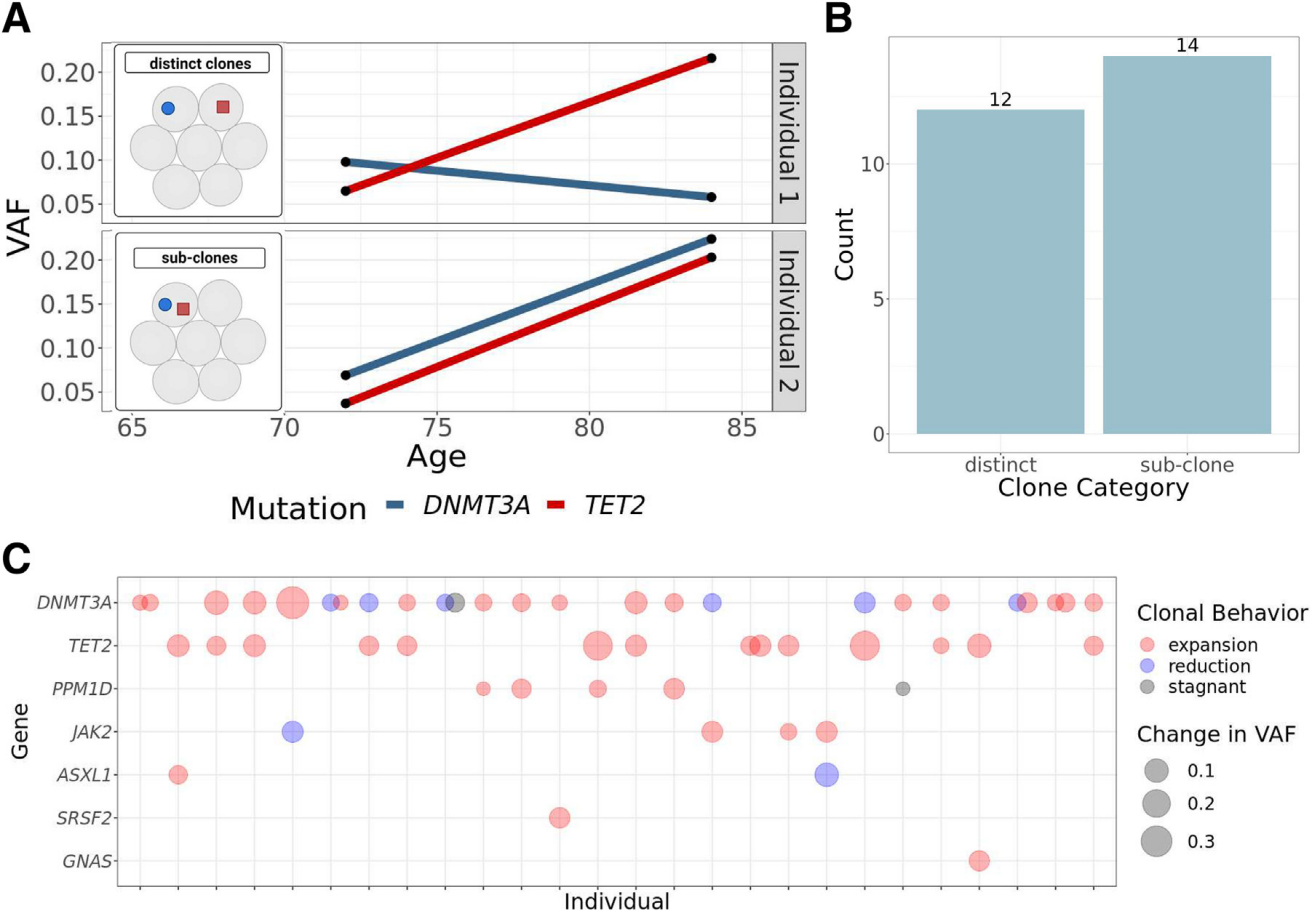
Clonal trajectories over time in individuals with >one driver mutation. **A:** In individuals with >one driver mutation, different clonal trajectories are possible. The mutations can be either in completely separate cells (*distinct clones*) or in the same cells (*sub-clones*). For distinct clones, different growth rates and clonal behavior would be expected, while sub-clones should show similar growth rates. In this hypothetical example, Individual 1 likely shows distinct clonal behavior, while Individual 2 likely shows sub-clonal behavior. **B:** Number of individuals in each sub-clone and distinct clone group. **C:** CHIP driver genes in each individual with two CHIP driver mutations. Each **vertical line** on the *x* axis represents one individual, and each point represents a CHIP clone. If an individual has both mutations on the same driver gene, the points are slightly overlapping.

In individuals with more than one driver mutation, it is possible that the mutations were in distinct cell populations (distinct clones) or that the two mutations co-occurred in the same cell populations (sub-clones), as shown in the hypothetical example in Figure 4A. Using clone trajectory, the most likely scenario was identified for individuals with two mutations (see Materials and Methods), and each person was classified into one of the two groups (distinct clones versus sub-clones). Of the 26 individuals with two mutations, 12 had distinct clones and 14 had sub-clones (Figure 4B). When the mean growth rate of each individual's fastest-growing clone was compared by group, the difference between the distinct and sub-clone groups was not significant (*P* = 0.20). When looking at the combinations of driver genes, *DNMT3A* mutations were most likely to remain stagnant or be reduced in the presence of an additional driver mutation compared to other drivers (Figure 4C). The co-occurrence of driver genes was significantly different than was expected by random chance while controlling for driver-gene prevalence in the cohort (χ^2^ = 322.03, *P* = 8.73 × 10^−11^). *PPM1D* and *DNMT3A* mutations co-occurred more frequently than expected (expected, 0.9/26; observed, 4/26; *P* = 1), as did *DNMT3A* and *TET2* mutations (expected, 2.8/26; observed, 8/26; *P* = 0.16), but these differences were not statistically significant.

### No Association between Demographic Factors and CHIP Clonal Expansion

Previous studies have suggested that demographic factors may contribute to CHIP prevalence, expansion rate, and risk for subsequent disease.^2^, ^9^ In particular, there has been much speculation about the potential role of obesity or other metabolic factors in CHIP expansion. However, no significant effects of height (*P* = 0.98), self-reported race (*P* = 0.94), ethnicity (*P* = 0.77), biological sex (*P* = 0.68), age (*P* = 0.63), or body mass index (*P* = 0.37) on clonal expansion rate across individuals in this cohort while controlling for driver-gene identity were detected (Supplemental Table S5).

## Discussion

This study presents a highly scalable and cost-efficient CHIP detection assay optimized for the detection of CHIP mutations in large-scale cohorts. As a demonstration of the utility of this targeted assay, a set of multi–time point blood samples from the BioVU biobank were profiled. Insights from this cohort showed the influence of driver gene, mutation type, mutation quantity, and demographic information on clonal expansion rate over time.

Although CHIP mutations can be detected using whole-genome/exome sequencing, the high cost and the complexity of the method are both not necessary to understand CHIP clonal dynamics. Other targeted methods, such as the TruSight myeloid sequencing panel (Illumina), also involve high cost and time-consuming protocols. The current targeted-gene panel approach is low-cost and is therefore much more practical for large-scale CHIP studies. Currently, the Vanderbilt Technologies for Advanced Genomics (VANTAGE) core laboratory performs this assay on a cost-recovery, fee-for-service basis for approximately US $8.40 per sample, which is approximately 30- to 50-fold less expensive than alternative technologies. Approximately 80,000 samples have been run in the past 12 months for investigators at 30 institutions across the United States and incorporated into multiple published reports.^14^^,^^25^, ^26^, ^27^ The key innovations of the assay include miniaturizing and identifying the optimal stoichiometry of specific DNA library preparation and capture components, and identifying a 44-Kb DNA target region capable of detecting >95% of CHIP mutations in the population. This assay can accurately identify CHIP mutations and quantify them effectively as they persist over time. Due to the deep sequencing coverage of the assay, clonal fractions of CHIP mutations below the typical 2% VAF cutoff were also detected. Although individuals with CHIP clones of this size are less likely to progress to disease, they are still at increased risks for blood cancer and heart failure.^28^^,^^29^ Further work is needed to fully characterize the clinical risk for these small clones, and will be made possible by assays like this CHIP assay with the power to detect small clones below the lower limit of detection of exome/genome sequencing.

The application of the CHIP assay to samples from the Vanderbilt BioVU participants revealed interesting observations about clonal behavior over time. In addition to this report, three other recent papers describe CHIP expansion rate in cohorts with multi–time point blood samples.^19^^,^^30^^,^^31^ These results are highly concordant with those from a previous study, showing that CHIP mutations are enriched in older individuals and that *DNMT3A* and *TET2* are the most common CHIP driver genes.^9^ In this cohort, *JAK2* and *PPM1D* mutations were the next two most common mutations, followed by *ASXL1*. *JAK2* mutations, that were enriched in this set of BioVU samples because *JAK2* p.V617F is directly genotyped on the BioVU MEGA array, and samples with predicted CHIP were intentionally included. Upon chart review, 56% of individuals with *PPM1D* mutations underwent chemotherapy, so the overrepresentation of *PPM1D* is likely related to the fact that BioVU is a tertiary hospital–based cohort.

Most individuals with CHIP/CCUS had a single driver mutation, but individuals with multiple driver mutations were also identified in the cohort. Concordant with previous work, *TET2* surpassed *DNMT3A* as the most common driver gene in individuals aged ≥75 years.^30^

An understanding of the factors that contribute to clonal expansion is important because faster-growing clones have been associated with a greater risk for disease.^18^^,^^28^^,^^32^ As in prior work, in this study, driver genes played a large role in determining the rate of clonal growth.^30^ However, the driver-gene mutation is not deterministic, and there is a significant level of individual-level heterogeneity. When focusing on specific driver mutations within the same gene in *DNMT3A,* no significant differences in mean growth rate between the two hotspots, R882C and R882H, were observed. Additionally, no significant differences were observed between loss-of-function and missense mutations, although the sample size of the loss-of-function group (*n* = 6) precluded robust inference. Future efforts with larger sample sizes or a meta-analysis across existing published data may power further analyses at the individual-mutation level.

The findings from this study add to the recent surprising observations in comparable cohorts,^19^^,^^30^ that CHIP clones do not universally grow over time but can also remain stagnant, or even shrink. While most clones grew, approximately 25% of clones displayed shrinkage, and reduction clones persisted even in individuals with only one CHIP clone. It is possible that these clones were reduced due to the presence of an additional CHIP/non-CHIP clone that is more fit,^31^ or that additional lifestyle/environmental factors influenced the clone. Within individuals with two driver mutations, *DNMT3A* clones were more likely than other driver genes to remain stagnant or be reduced over time, which is consistent with their slow growth rate on average. The vast majority of individuals with two CHIP clones had at least one clone that was expanding. Further studies are needed to better characterize differences in clonal-behavior trajectories.

In individuals with two CHIP driver mutations, it is possible that either those mutations occurred in distinct subpopulations of cells or that they co-occurred in sub-clonal populations. Single–time point data do not allow for any distinction between these possibilities because the VAF of the mutations would be the same in both cases. However, multi–time point cohorts provide additional information about clonal behavior over time that can provide insight into which of these scenarios is most likely. In this cohort, there was nearly a 50–50 split in distinct clone versus sub-clone individuals, indicating that both possibilities occurred in individuals with CHIP/CCUS. A significant difference in mean growth rate of the fastest-growing clone between these two groups was not observed. The distribution of driver genes was not random, and combinations of *DNMT3A* + *PPM1D* and *DNMT3A* + *TET2* mutations were particularly enriched, even when controlling for their overall prevalence in the cohort. There may be underlying biology underlying the co-occurrence of these mutations, but this could also have been an artifact of the hospital-based cohort. Future studies are required to resolve whether these two scenarios confer differential disease risk.

While age and body mass index have been previously associated with increased CHIP prevalence,^33^ and metabolic factors may increase CHIP expansion rate,^34^^,^^35^ in this study, participants' characteristics did not have a significant effect on clonal growth rate. This finding is concordant with previous work that also did not detect any associations between clonal expansion rate and race, ethnicity, or body mass index.^19^ Larger-scale studies are needed to identify the genetic and environmental influences on the rate of clonal expansion.

This work had limitations that are important to consider. First, although this study was one of the largest multi–time point longitudinal studies of CHIP mutations, the cohort sample size was small in absolute terms, meaning that individual-level outliers had the potential to influence the general conclusions of the study. Second, the range of time between blood draws represented in the study was from 6 months to 12 years. However, the process of calculating clonal growth rate (see Materials and Methods) accounted for both the starting VAF and the time between blood draws. Third, the CHIP assay was designed to capture >95% of CHIP mutations, meaning that it was unable to capture the entire range of CHIP mutations and could not detect larger structural changes (eg, large-scale chromosomal deletions or loss of heterozygosity) that can also cause clonal expansion. Efforts are underway to develop a second-generation assay to detect larger-scale alterations. Fourth, although the assay achieved deep sequencing depth, expansion/reduction could still have been affected by biological factors, such as the relative proportion of myeloid cells at the time of blood draw.

In summary, this study presents a cost-effective and accurate tool to call CHIP mutations in large-scale cohorts. The application of this tool to a multi–time point CHIP/CCUS cohort in the Vanderbilt BioVU biobank revealed important observations, such as the influence of driver gene on clonal growth and the various clonal-behavior trajectories observed across driver genes. Use of this assay in >80,000 samples by investigators around the world has already enabled large-scale studies to characterize the influence of CHIP on health. This highly cost-effective tool is expected to be of particular utility in the large-scale screening that will be required for identifying participants for future CHIP clinical trials.

## Disclosure Statement

M.R.S. has received honoraria for advisory board membership or consultancy from Bristol Myers Squibb, CTI, Forma, Geron, GlaxoSmithKline/Sierra Oncology, Karyopharm, Ryvu Therapeutics, and Taiho Pharmaceutical; has received research funding from ALX Oncology, Astex Pharmaceuticals, Incyte Corporation, Takeda, and TG Therapeutics; holds equity in Empath Biosciences, Karyopharm, and Ryvu Therapeutics; and has been reimbursed for travel expenses by Astex. A.G.B. has received honoraria for advisory board membership from, and holds equity in, TenSixteen Bio.

## Supplemental Data

Supplemental Table S5

Supplemental Figure S1 Flow chart of inclusion criteria for CHIP analyses. A subset of individuals from the Vanderbilt BioVU cohort (approximately 300,000) have more than one blood sample, forming the BioVU multi–time point (MTP) cohort (approximately 30,000). A portion of those individuals (*n* = 456) were sequenced on the CHIP assay based on predicted CHIP. CHIP was predicted using the genotyping data from the BioVU MEGA array data. In 283 of those individuals, CHIP was not detected. In the remaining 173, patient charts were examined for the presence of blood cancer. If blood cancer was present in the record, the data from that individual were excluded. A total of 101 individuals with CHIP remained and the data from those individuals were used for further analyses.

Supplemental Figure S2 Prevalence of CHIP driver genes in the TOPMed cohort (data from Supplemental Table S3 of Bick et al^9^). Bar plot and pie chart showing the distribution of CHIP driver genes across the TOPMed cohort (4938 CHIP mutations in 4229 individuals). More than 75% of mutations were in either *DNMT3A, TET2,* or *ASXL1*.

Supplemental Figure S3 Individual clonal dynamics over time. Plot showing the clonal behavior in each individual with two CHIP driver mutations. Each **line** represents one clone, and each small plot represents an individual from the cohort. Facets on the left are categorized as sub-clonal, while facets on the right are categorized as distinct clones.

Supplemental Figure S4 Limit of detection test for CHIP detection assay. Results of a limiting dilution experiment in which a DNA sample with known genotype was combined at serial fixed ratios with a second sample of known genotype. Each color represents a distinct sample, and the correlation line is displayed to show the correlation between observed and expected allele fraction. This method robustly detects variants present in >1% of DNA. Beneath this 1% threshold, variants down to approximately 0.1% allele fraction are detected, but with less accuracy for the estimated allele fraction.
