# Supplementary material for: Cost-Effective and Scalable Clonal Hematopoiesis Assay Provides Insight into Clonal Dynamics
Source: J Mol Diagn. 2024 Jul;26(7):563–73. doi: 10.1016/j.jmoldx.2024.03.007 (PMC11536471; doi:10.1016/j.jmoldx.2024.03.007)
Supplement: Supplemental Table S1 [file mmc1.docx]

**Supplemental Table 1: Descriptive statistics on study cohort and demographic information.**

| **Cohort** | **Number of Individuals** | **Mean Age (Timepoint 1)** | **Mean Age (Timepoint 2)** | **Biological Sex** | **Self-Reported Race** |
| --- | --- | --- | --- | --- | --- |
| BioVU MTP w/Predicted CHIP | 456 | 52.5 years  (SD: 19 years) | 57 years (SD: 19 years) | 60% female | 81% Caucasian  15% African American  1% Asian |
| BioVU MTP without CHIP Mutation | 283 | 47 years (SD: 19 years) | 52 years (SD: 19 years) | 59% female | 77% Caucasian  19% African American  2% Asian |
| BioVU MTP w/CHIP mutation & Blood Cancer | 72 | 60 years (SD: 17 years) | 64 years (SD: 17 years) | 63% female | 93% Caucasian  4% African American |
| BioVU MTP with CHIP/CCUS | 101 | 64 years (SD: 14 years) | 69 years (SD: 14 years) | 64% female | 88% Caucasian  10% African American |
