# Supplementary material for: Cost-Effective and Scalable Clonal Hematopoiesis Assay Provides Insight into Clonal Dynamics
Source: J Mol Diagn. 2024 Jul;26(7):563–73. doi: 10.1016/j.jmoldx.2024.03.007 (PMC11536471; doi:10.1016/j.jmoldx.2024.03.007)
Supplement: Supplemental Table S2 [file mmc2.docx]

**Supplemental Table 2: Cost breakdown of CHIP Sequencing Assay**

| **Resource** | **Cost** | **Hours** | **Unit** |
| --- | --- | --- | --- |
| Twist Library Preparation Kit, Enzymatic Fragmentation, 96 rxn | $2.25 | 1.5 | plate |
| Twist Universal Adapter System-Universal Adapters and UDI Primers SET A,B,C,D(96) | $0.55 |  |  |
| Microseal B adhesive seals | $0.39 |  |  |
| Eppendorf 96-well twin.tec plates, Clear; Semi-skirted | $0.78 |  |  |
| Ethanol - 200 proof (absolute) | $0.30 |  |  |
| Reservoirs, 25 ml(CS) | $0.50 |  |  |
| Agencourt AMPure Beads XP kit, 60 ml | $0.40 |  |  |
| Nitrile Gloves | $0.29 |  |  |
| Beckman-P50 Sterile Barrier (pink) | $0.17 |  |  |
|  |  |  |  |
| **Resource** | **Cost** | **Hours** | **Unit** |
| Twist Custom panel_Bick_Clonal_Hematapoesis_v2 (96 probes) | $0.99 | 1.5 | plate |
| Twist Universal Blocker(96 rxn) | $0.12 |  |  |
| Twist binding and purification beads(96 rxn) | $0.12 |  |  |
| Twist Hybridization and Wash Kit(96 rxn) | $0.07 |  |  |
| Qubit dsDNA HS High Sensitivity Assay Kit | $0.06 |  |  |
| Qubit Assay Tubes | $0.02 |  |  |
| Eppendorf Lo-Bind 1.5 ml tube | $0.13 |  |  |
| Reservoirs, 25 ml(CS) | $0.02 |  |  |
| Rainin Pipette Tips, P1000 | $0.04 |  |  |
| Rainin Pipette Tips, P200 | $0.08 |  |  |
|  |  |  |  |
|  | **Total Reagent and Consumable cost** | $7.28 |  |
|  | **Total Tech Hours** | $0.16 |  |
|  | **Other (service contracts, depreciation, etc.)** | $1.44 |  |
|  |  |  |  |
|  | **Total per sample** | $8.88 |  |
