# Supplementary material for: Cost-Effective and Scalable Clonal Hematopoiesis Assay Provides Insight into Clonal Dynamics
Source: J Mol Diagn. 2024 Jul;26(7):563–73. doi: 10.1016/j.jmoldx.2024.03.007 (PMC11536471; doi:10.1016/j.jmoldx.2024.03.007)
Supplement: Supplemental Table S4 [file mmc4.docx]

**Supplemental Table 4: CHIP mutations present in cohort.**

| ID | Gene | NonsynOI | CHR | POS | Age_A | Age_B | AF_A | AF_B | AF_Diff |
| --- | --- | --- | --- | --- | --- | --- | --- | --- | --- |
| 1 | *TET2* | F775Sfs*38 | chr4 | 1.05E+08 | 55 | 64 | 0.001 | 0.033 | 0.032 |
| 2 | *DNMT3A* | M761V | chr2 | 25240343 | 72 | 74 | 0.02 | 0.023 | 0.003 |
| 2 | *DNMT3A* | splicing | chr2 | 25239129 | 72 | 74 | 0.0125 | 0.022 | 0.0095 |
| 3 | *DNMT3A* | R736C | chr2 | 25240418 | 54 | 56 | 0.143 | 0.178 | 0.035 |
| 4 | *ASXL1* | E948Gfs*7 | chr20 | 32435552 | 81 | 85 | 0.317 | 0.342 | 0.025 |
| 4 | *TET2* | splicing | chr4 | 1.05E+08 | 81 | 85 | 0.262 | 0.329 | 0.067 |
| 5 | *DNMT3A* | R882C | chr2 | 25234374 | 61 | 63 | 0.056 | 0.052 | -0.004 |
| 6 | *PPM1D* | T529Mfs*13 | chr17 | 60663311 | 62 | 70 | 0.001 | 0.27 | 0.269 |
| 7 | *TET2* | P1962T | chr4 | 1.05E+08 | 32 | 41 | 0.0008 | 0.024 | 0.0232 |
| 8 | *TET2* | F854Lfs*19 | chr4 | 1.05E+08 | 79 | 82 | 0.099 | 0.263 | 0.164 |
| 9 | *DNMT3A* | R882C | chr2 | 25234374 | 91 | 94 | 0.11 | 0.096 | -0.014 |
| 10 | *DNMT3A* | R882C | chr2 | 25234374 | 67 | 68 | 0.368 | 0.342 | -0.026 |
| 11 | *DNMT3A* | R882C | chr2 | 25234374 | 42 | 53 | 0.203 | 0.306 | 0.103 |
| 11 | *TET2* | D1844Vfs*43 | chr4 | 1.05E+08 | 42 | 53 | 0.016 | 0.045 | 0.029 |
| 12 | *JAK2* | V617F | chr9 | 5073770 | 19 | 26 | 0.049 | 0.002595 | -0.0464 |
| 13 | *DNMT3A* | R882C | chr2 | 25234374 | 53 | 56 | 0.269 | 0.356 | 0.087 |
| 14 | *DNMT3A* | R882H | chr2 | 25234373 | 35 | 46 | 0.101 | 0.183 | 0.082 |
| 14 | *TET2* | P363Lfs*9 | chr4 | 1.05E+08 | 35 | 46 | 0.004 | 0.074 | 0.07 |
| 15 | *DNMT3A* | R882C | chr2 | 25234374 | 52 | 56 | 0.062 | 0.093 | 0.031 |
| 16 | *JAK2* | V617F | chr9 | 5073770 | 48 | 56 | 0.024 | 0.041 | 0.017 |
| 17 | *DNMT3A* | Q573X | chr2 | 25244289 | 81 | 82 | 0.067 | 0.042 | -0.025 |
| 18 | *DNMT3A* | N717S | chr2 | 25240663 | 76 | 78 | 0.021 | 0.008 | -0.013 |
| 19 | *JAK2* | V617F | chr9 | 5073770 | 70 | 78 | 0.071 | 0.013 | -0.058 |
| 19 | *DNMT3A* | R729W | chr2 | 25240439 | 70 | 78 | 0.03 | 0.376 | 0.346 |
| 20 | *DNMT3A* | splicing | chr2 | 25244340 | 53 | 56 | 0.02 | 0.025 | 0.005 |
| 21 | *DNMT3A* | R882C | chr2 | 25234374 | 74 | 78 | 0.076 | 0.073 | -0.003 |
| 21 | *DNMT3A* | Y735C | chr2 | 25240420 | 74 | 78 | 0.037 | 0.038 | 0.001 |
| 21 | *DNMT3A* | Y533C | chr2 | 25244609 | 74 | 78 | 0.008 | 0.02 | 0.012 |
| 22 | *DNMT3A* | R882H | chr2 | 25234373 | 79 | 85 | 0.14 | 0.178 | 0.038 |
| 23 | *DNMT3A* | R882H | chr2 | 25234373 | 55 | 60 | 0.137 | 0.125 | -0.012 |
| 23 | *DNMT3A* | R771X | chr2 | 25240313 | 55 | 60 | 0.018 | 0.02 | 0.002 |
| 24 | *DNMT3A* | R749C | chr2 | 25240379 | 44 | 46 | 0.145 | 0.123 | -0.022 |
| 24 | *TET2* | C1135Y | chr4 | 1.05E+08 | 44 | 46 | 0.097 | 0.131 | 0.034 |
| 25 | *TET2* | S1607Ffs*7 | chr4 | 1.05E+08 | 44 | 47 | 0.001 | 0.036 | 0.035 |
| 25 | *DNMT3A* | R882H | chr2 | 25234373 | 44 | 47 | 0.035 | 0.044 | 0.009 |
| 26 | *JAK2* | V617F | chr9 | 5073770 | 72 | 77 | 0.064 | 0.074 | 0.01 |
| 27 | *DNMT3A* | R882C | chr2 | 25234374 | 66 | 75 | 0.112 | 0.198 | 0.086 |
| 28 | *DNMT3A* | R882C | chr2 | 25234374 | 44 | 48 | 0.032 | 0.064 | 0.032 |
| 29 | *DNMT3A* | R882H | chr2 | 25234373 | 77 | 88 | 0.025 | 0.037 | 0.012 |
| 29 | *SF3B1* | T663I | chr2 | 1.97E+08 | 77 | 88 | 0.001 | 0.032 | 0.031 |
| 29 | *TET2* | A1379V | chr4 | 1.05E+08 | 77 | 88 | 0.001 | 0.051 | 0.05 |
| 30 | *TET2* | R1161G | chr4 | 1.05E+08 | 60 | 68 | 0.022 | 0.001 | -0.021 |
| 30 | *TET2* | S1582Qfs*16 | chr4 | 1.05E+08 | 60 | 68 | 0.142 | 0.605 | 0.463 |
| 30 | *DNMT3A* | R882C | chr2 | 25234374 | 57 | 60 | 0.007 | 0.037 | 0.03 |
| 31 | *DNMT3A* | R882H | chr2 | 25234373 | 59 | 62 | 0.015 | 0.033 | 0.018 |
| 32 | *DNMT3A* | Q374Rfs*33 | chr2 | 25247052 | 63 | 70 | 0.024 | 0.0127 | -0.0113 |
| 32 | *DNMT3A* | R882C | chr2 | 25234374 | 63 | 70 | 0.232 | 0.261 | 0.029 |
| 33 | *JAK2* | V617F | chr9 | 5073770 | 65 | 73 | 0.108 | 0.06 | -0.048 |
| 33 | *GNB1* | K57E | chr1 | 1815790 | 65 | 73 | 0.046 | 0.187 | 0.141 |
| 33 | *DNMT3A* | I705Mfs*74 | chr2 | 25240698 | 65 | 73 | 0.039 | 0.203 | 0.164 |
| 34 | *TET2* | H222Wfs*2 | chr4 | 1.05E+08 | 47 | 52 | 0.198 | 0.218 | 0.02 |
| 35 | *DNMT3A* | Q846X | chr2 | 25235768 | 65 | 71 | 0.022 | 0.015 | -0.007 |
| 36 | *DNMT3A* | R882C | chr2 | 25234374 | 56 | 61 | 0.15 | 0.211 | 0.061 |
| 37 | *JAK2* | V617F | chr9 | 5073770 | 34 | 37 | 0.004 | 0.023 | 0.019 |
| 38 | *ASXL1* | E635Rfs*15 | chr20 | 32434600 | 55 | 61 | 0.001 | 0.05 | 0.049 |
| 38 | *DNMT3A* | R882H | chr2 | 25234373 | 55 | 61 | 0.026 | 0.087 | 0.061 |
| 38 | *TET2* | P1092Qfs*14 | chr4 | 1.05E+08 | 55 | 61 | 0.027 | 0.09 | 0.063 |
| 39 | *JAK2* | V617F | chr9 | 5073770 | 55 | 59 | 0.029 | 0.004132 | -0.02487 |
| 40 | *CBL* | C404Y | chr11 | 1.19E+08 | 63 | 65 | 0.026 | 0.01325 | -0.01275 |
| 41 | *DNMT3A* | R882H | chr2 | 25234373 | 79 | 80 | 0.097 | 0.079 | -0.018 |
| 42 | *DNMT3A* | W753R | chr2 | 25240367 | 50 | 58 | 0.001 | 0.025 | 0.024 |
| 43 | *JAK2* | V617F | chr9 | 5073770 | 55 | 56 | 0.001 | 0.049 | 0.048 |
| 44 | *DNMT3A* | R882C | chr2 | 25234374 | 75 | 78 | 0.022 | 0.033 | 0.011 |
| 44 | *PPM1D* | W427X | chr17 | 60663015 | 75 | 78 | 0.019 | 0.02 | 0.001 |
| 45 | *GNAS* | R201H | chr20 | 58909366 | 60 | 67 | 0.015 | 0.029 | 0.014 |
| 46 | *DNMT3A* | R882H | chr2 | 25234373 | 73 | 77 | 0.045 | 0.063 | 0.018 |
| 46 | *PPM1D* | M515Lfs*4 | chr17 | 60663277 | 73 | 77 | 0.026 | 0.059 | 0.033 |
| 47 | *DNMT3A* | W440Vfs*5 | chr2 | 25246271 | 52 | 61 | 0.025 | 0.025 | 0 |
| 48 | *TET2* | S1848X | chr4 | 1.05E+08 | 88 | 92 | 0.024 | 0.029 | 0.005 |
| 48 | *SF3B1* | K700E | chr2 | 1.97E+08 | 88 | 92 | 0.028 | 0.033 | 0.005 |
| 48 | *TET2* | Y1255X | chr4 | 1.05E+08 | 88 | 92 | 0.047 | 0.055 | 0.008 |
| 48 | *TET2* | K1001Mfs*6 | chr4 | 1.05E+08 | 88 | 92 | 0.001 | 0.027 | 0.026 |
| 49 | *DNMT3A* | R882C | chr2 | 25234374 | 54 | 59 | 0.015 | 0.024 | 0.009 |
| 50 | *DNMT3A* | R882H | chr2 | 25234373 | 63 | 64 | 0.143 | 0.183 | 0.04 |
| 51 | *DNMT3A* | Y735C | chr2 | 25240420 | 90 | 92 | 0.03 | 0.034 | 0.004 |
| 51 | *SRSF2* | P95L | chr17 | 76736877 | 90 | 92 | 0.096 | 0.145 | 0.049 |
| 52 | *PPM1D* | N512Ifs*2 | chr17 | 60663263 | 67 | 70 | 0.069 | 0.1 | 0.031 |
| 53 | *JAK2* | V617F | chr9 | 5073770 | 51 | 61 | 0.052 | 0.052 | 0 |
| 54 | *PPM1D* | R552X | chr17 | 60663388 | 77 | 83 | 0.01 | 0.023 | 0.013 |
| 54 | *TET2* | L1360R | chr4 | 1.05E+08 | 77 | 83 | 0.014 | 0.254 | 0.24 |
| 55 | *DNMT3A* | R882H | chr2 | 25234373 | 43 | 50 | 0.027 | 0.051 | 0.024 |
| 56 | *DNMT3A* | R882C | chr2 | 25234374 | 62 | 65 | 0.329 | 0.314 | -0.015 |
| 57 | *DNMT3A* | R771Q | chr2 | 25240312 | 64 | 66 | 0.02 | 0.021 | 0.001 |
| 58 | *TET2* | I1873T | chr4 | 1.05E+08 | 57 | 61 | 0.035 | 0.092 | 0.057 |
| 58 | *DNMT3A* | R882C | chr2 | 25234374 | 57 | 61 | 0.076 | 0.149 | 0.073 |
| 59 | *GNAS* | R201C | chr20 | 58909365 | 81 | 85 | 0.095 | 0.156 | 0.061 |
| 60 | *DNMT3A* | R771X | chr2 | 25240313 | 73 | 75 | 0.042 | 0.059 | 0.017 |
| 61 | *DNMT3A* | R882H | chr2 | 25234373 | 37 | 40 | 0.028 | 0.035 | 0.007 |
| 62 | *SF3B1* | R625L | chr2 | 1.97E+08 | 84 | 88 | 0.007 | 0.024 | 0.017 |
| 63 | *DNMT3A* | F755S | chr2 | 25240360 | 86 | 90 | 0.01 | 0.02 | 0.01 |
| 64 | *DNMT3A* | R771X | chr2 | 25240313 | 73 | 79 | 0.023 | 0.01 | -0.013 |
| 64 | *DNMT3A* | R882H | chr2 | 25234373 | 73 | 79 | 0.132 | 0.18 | 0.048 |
| 64 | *TET2* | splicing | chr4 | 1.05E+08 | 73 | 79 | 0.001 | 0.022 | 0.021 |
| 65 | *ASXL1* | Q910Tfs*14 | chr20 | 32435436 | 65 | 66 | 0.077 | 0.078 | 0.001 |
| 66 | *JAK2* | V617F | chr9 | 5073770 | 47 | 55 | 0.033 | 0.029 | -0.004 |
| 67 | *PPM1D* | R552X | chr17 | 60663388 | 65 | 68 | 0.335 | 0.448 | 0.113 |
| 68 | *DNMT3A* | R882H | chr2 | 25234373 | 78 | 81 | 0.061 | 0.104 | 0.043 |
| 69 | *DNMT3A* | V657M | chr2 | 25241675 | 48 | 52 | 0.049 | 0.026 | -0.023 |
| 70 | *DNMT3A* | R882H | chr2 | 25234373 | 61 | 71 | 0.06 | 0.055 | -0.005 |
| 71 | *DNMT3A* | R882C | chr2 | 25234374 | 61 | 68 | 0.293 | 0.319 | 0.026 |
| 72 | *PPM1D* | N460Mfs*5 | chr17 | 60663111 | 36 | 46 | 0.001 | 0.051 | 0.05 |
| 72 | *DNMT3A* | S179Qfs*39 | chr2 | 25275045 | 36 | 46 | 0.001 | 0.02 | 0.019 |
| 73 | *DNMT3A* | C710Y | chr2 | 25240684 | 64 | 70 | 0.022 | 0.031 | 0.009 |
| 74 | *DNMT3A* | S770L | chr2 | 25240315 | 61 | 70 | 0.04 | 0.02 | -0.02 |
| 74 | *JAK2* | V617F | chr9 | 5073770 | 61 | 70 | 0.11 | 0.164 | 0.054 |
| 75 | *TET2* | R550X | chr4 | 1.05E+08 | 72 | 84 | 0.026 | 0.058 | 0.032 |
| 75 | *TET2* | R1214Q | chr4 | 1.05E+08 | 72 | 84 | 0.065 | 0.123 | 0.058 |
| 76 | *JAK2* | V617F | chr9 | 5073770 | 53 | 59 | 0.017 | 0.026 | 0.009 |
| 76 | *TET2* | A1341P | chr4 | 1.05E+08 | 53 | 59 | 0.005 | 0.054 | 0.049 |
| 77 | *DNMT3A* | R882C | chr2 | 25234374 | 82 | 87 | 0.094 | 0.115 | 0.021 |
| 78 | *ASXL1* | R965X | chr20 | 32435605 | 72 | 74 | 0.128 | 0.027 | -0.101 |
| 78 | *JAK2* | V617F | chr9 | 5073770 | 72 | 74 | 0.099 | 0.154 | 0.055 |
| 79 | *TET2* | V218Wfs*32 | chr4 | 1.05E+08 | 78 | 80 | 0.158 | 0.248 | 0.09 |
| 80 | *DNMT3A* | R882C | chr2 | 25234374 | 51 | 59 | 0.035 | 0.079 | 0.044 |
| 81 | *DNMT3A* | E774K | chr2 | 25240304 | 78 | 88 | 0.098 | 0.047 | -0.051 |
| 81 | *TET2* | T1066Lfs*16 | chr4 | 1.05E+08 | 78 | 88 | 0.069 | 0.316 | 0.247 |
| 82 | *PPM1D* | R572X | chr17 | 60663448 | 50 | 54 | 0.022 | 0.023 | 0.001 |
| 82 | *DNMT3A* | R882H | chr2 | 25234373 | 50 | 54 | 0.065 | 0.074 | 0.009 |
| 83 | *TET2* | R1440Tfs*38 | chr4 | 1.05E+08 | 79 | 80 | 0.358 | 0.443 | 0.085 |
| 84 | *DNMT3A* | R882H | chr2 | 25234373 | 52 | 54 | 0.13 | 0.138 | 0.008 |
| 84 | *TET2* | K1439Nfs*9 | chr4 | 1.05E+08 | 52 | 54 | 0.015 | 0.02 | 0.005 |
| 85 | *ASXL1* | G646Wfs*12 | chr20 | 32434638 | 62 | 68 | 0.079 | 0.214 | 0.135 |
| 86 | *GNAS* | R201H | chr20 | 58909366 | 76 | 83 | 0.001 | 0.046 | 0.045 |
| 86 | *TET2* | Q548X | chr4 | 1.05E+08 | 76 | 83 | 0.016 | 0.115 | 0.099 |
| 87 | *DNMT3A* | G707D | chr2 | 25240693 | 60 | 68 | 0.038 | 0.025 | -0.013 |
| 87 | *DNMT3A* | T251Pfs*61 | chr2 | 25248129 | 60 | 68 | 0.001 | 0.04 | 0.039 |
| 88 | *ASXL1* | R404X | chr20 | 32433408 | 67 | 68 | 0.052 | 0.083 | 0.031 |
| 89 | *DNMT3A* | R882H | chr2 | 25234373 | 58 | 66 | 0.199 | 0.203 | 0.004 |
| 90 | *TP53* | R175H | chr17 | 7675088 | 54 | 65 | 0.098 | 0.163 | 0.065 |
| 91 | *DNMT3A* | W753R | chr2 | 25240367 | 69 | 78 | 0.034 | 0.042 | 0.008 |
| 91 | *DNMT3A* | R635W | chr2 | 25243931 | 69 | 78 | 0.005 | 0.033 | 0.028 |
| 92 | *TET2* | Q744X | chr4 | 1.05E+08 | 76 | 84 | 0.03 | 0.039 | 0.009 |
| 93 | *PPM1D* | E472X | chr17 | 60663148 | 73 | 78 | 0.085 | 0.091 | 0.006 |
| 93 | *DNMT3A* | R882H | chr2 | 25234373 | 73 | 78 | 0.367 | 0.4 | 0.033 |
| 93 | *PPM1D* | L538X | chr17 | 60663347 | 73 | 78 | 0.096 | 0.178 | 0.082 |
| 93 | *TET2* | L1816Yfs*4 | chr4 | 1.05E+08 | 73 | 78 | 0.064 | 0.18 | 0.116 |
| 94 | *DNMT3A* | E283X | chr2 | 25248045 | 63 | 66 | 0.058 | 0.06 | 0.002 |
| 95 | *TET2* | Q913X | chr4 | 1.05E+08 | 81 | 85 | 0.033 | 0.032 | -0.001 |
| 96 | *DNMT3A* | E863Kfs*18 | chr2 | 25235718 | 68 | 70 | 0.072 | 0.088 | 0.016 |
| 96 | *TET2* | G1860E | chr4 | 1.05E+08 | 68 | 70 | 0.065 | 0.095 | 0.03 |
| 97 | *DNMT3A* | R882H | chr2 | 25234373 | 51 | 57 | 0.277 | 0.285 | 0.008 |
| 98 | *SF3B1* | E622D | chr2 | 1.97E+08 | 66 | 68 | 0.037 | 0.081 | 0.044 |
| 99 | *DNMT3A* | R882H | chr2 | 25234373 | 69 | 78 | 0.18 | 0.303 | 0.123 |
| 100 | *TET2* | Q1553X | chr4 | 1.05E+08 | 56 | 60 | 0.014 | 0.085 | 0.071 |
| 101 | *DNMT3A* | A398Pfs*9 | chr2 | 25246707 | 70 | 71 | 0.088 | 0.109 | 0.021 |
