# Supplementary material for: Cost-Effective and Scalable Clonal Hematopoiesis Assay Provides Insight into Clonal Dynamics
Source: J Mol Diagn. 2024 Jul;26(7):563–73. doi: 10.1016/j.jmoldx.2024.03.007 (PMC11536471; doi:10.1016/j.jmoldx.2024.03.007)
Supplement: Supplemental Table S5 [file mmc5.docx]

**Supplemental Table 5: Regression analysis of clonal growth rate with participant characteristics.**

| **Participant Characteristic** | **Beta Value** | **95% CI** | **P-Value** |
| --- | --- | --- | --- |
| Age at Timepoint 1 | -0.003 | [-0.02, 0.01] | 0.63 |
| Biological Sex | -0.09 | [-0.53, 0.34] | 0.68 |
| Self-reported Race | -0.09 | [-2.37, 2.19] | 0.94 |
| Ethnicity | 0.36 | [-2.04, 2.76] | 0.77 |
| BMI | 0.02 | [-0.02, 0.05] | 0.37 |
| Height | -0.0003 | [-0.02, 0.02] | 0.98 |
