# Supplementary material for: Cost-Effective and Scalable Clonal Hematopoiesis Assay Provides Insight into Clonal Dynamics
Source: J Mol Diagn. 2024 Jul;26(7):563–73. doi: 10.1016/j.jmoldx.2024.03.007 (PMC11536471; doi:10.1016/j.jmoldx.2024.03.007)
Supplement: Supplemental Figure S1 — Flow chart of inclusion criteria for CHIP analyses. A subset of individuals from the Vanderbilt BioVU cohort (approximately 300,000) have more than one blood sample, forming the BioVU multi–time point (MTP) cohort (approximately 30,000). A portion of those individuals (n = 456) were sequenced on the CHIP assay based on predicted CHIP. CHIP was predicted using the genotyping data from the BioVU MEGA array data. In 283 of those individuals, CHIP was not detected. In the remaining 173, patient charts were examined for the presence of blood cancer. If blood cancer was present in the record, the data from that individual were excluded. A total of 101 individuals with CHIP remained and the data from those individuals were used for further analyses. [file mmc6.pdf]

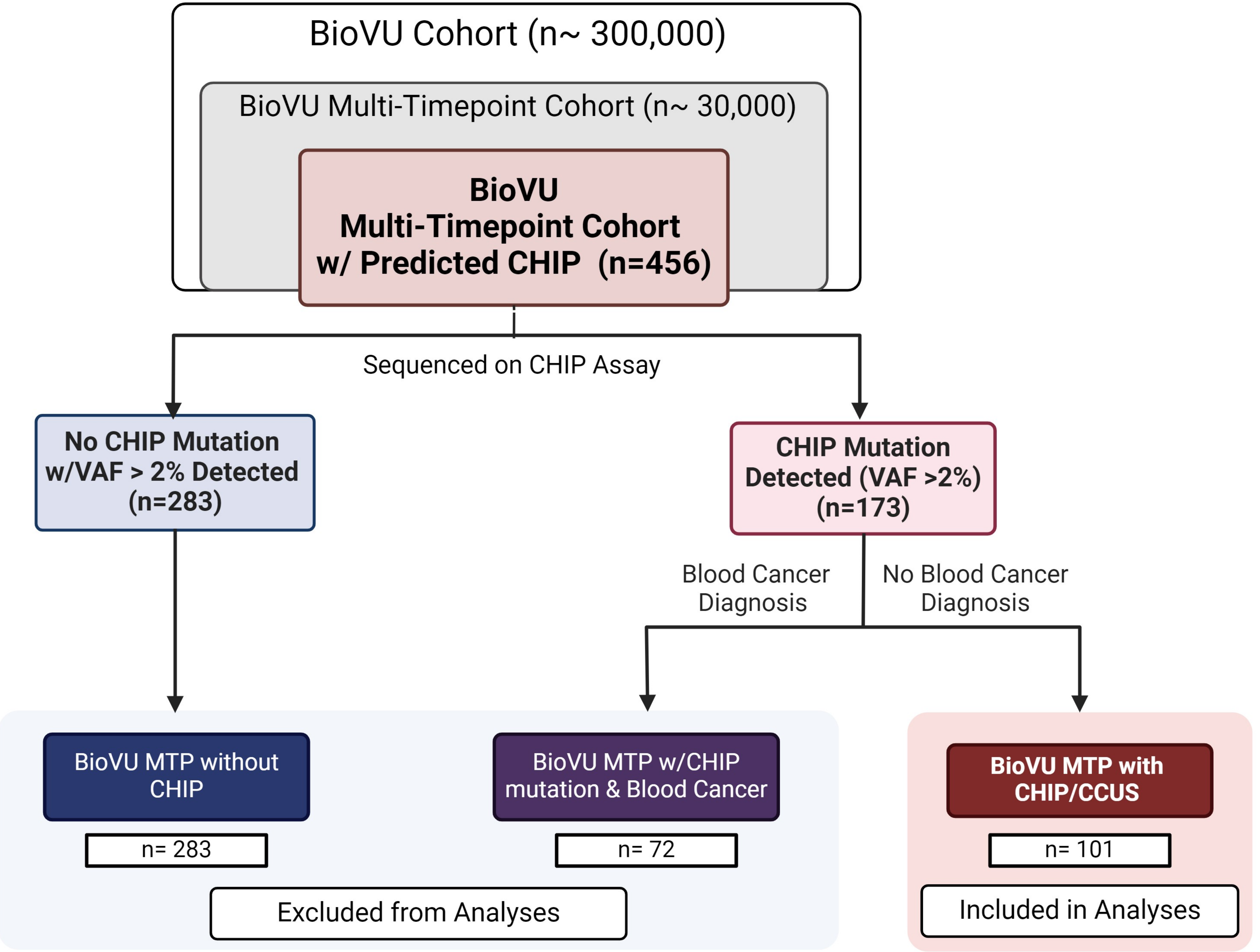
BioVU Cohort (n~ 300,000)
BioVU Multi-Timepoint Cohort (n~ 30,000)
BioVU Multi-Timepoint Cohort w/ Predicted CHIP (n=456)
Sequenced on CHIP Assay
No CHIP Mutation w/VAF > 2% Detected (n=283)
CHIP Mutation Detected (VAF >2%) (n=173)
Blood Cancer Diagnosis
No Blood Cancer Diagnosis
BioVU MTP without CHIP
n= 283
BioVU MTP w/CHIP mutation & Blood Cancer
n= 72
Excluded from Analyses
BioVU MTP with CHIP/CCUS
n= 101
Included in Analyses
