# Supplementary figures and images for: Cost-Effective and Scalable Clonal Hematopoiesis Assay Provides Insight into Clonal Dynamics
Source: J Mol Diagn. 2024 Jul;26(7):563–73. doi: 10.1016/j.jmoldx.2024.03.007 (PMC11536471; doi:10.1016/j.jmoldx.2024.03.007)

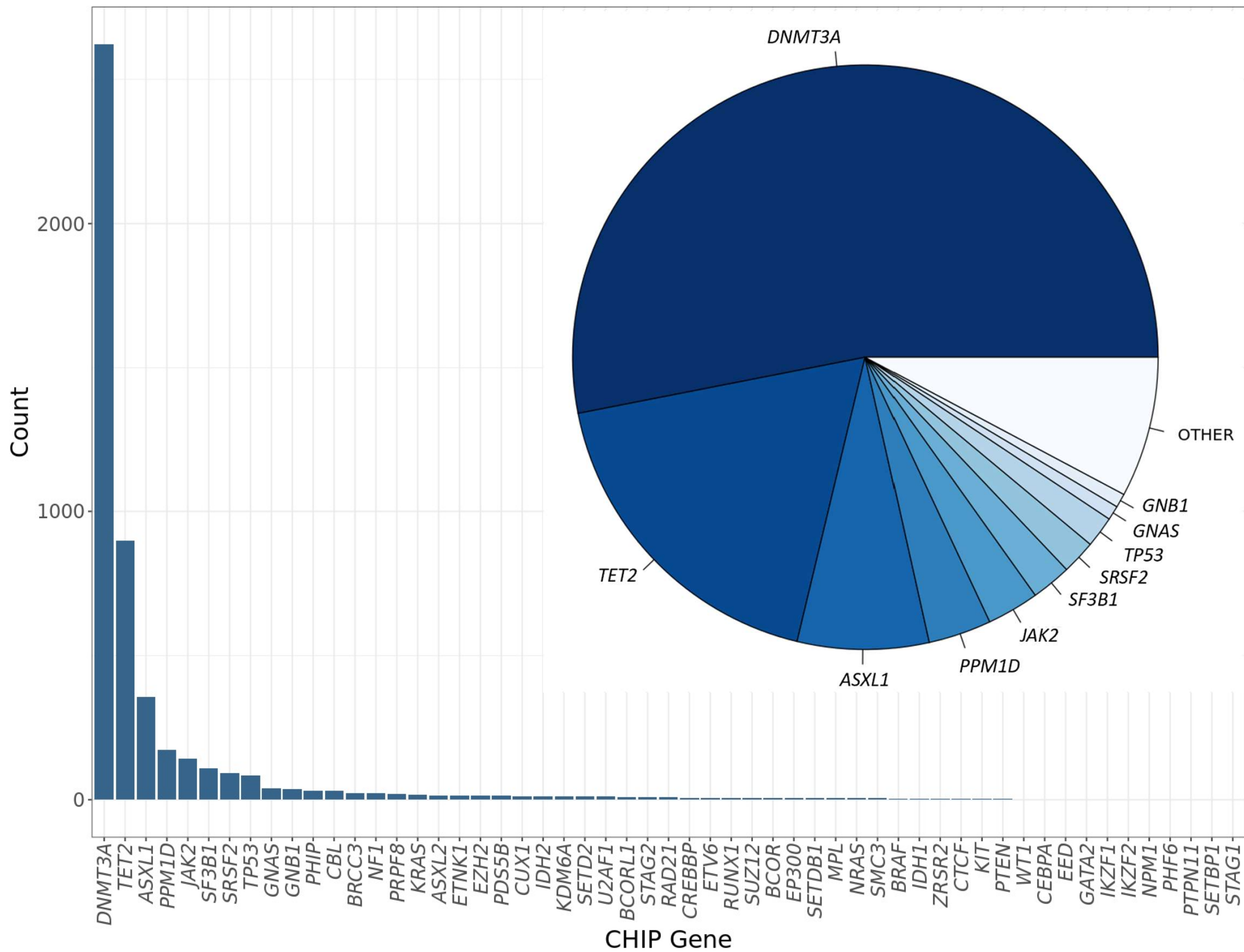

Supplement: Supplemental Figure S2 — Prevalence of CHIP driver genes in the TOPMed cohort (data from Supplemental Table S3 of Bick et al9). Bar plot and pie chart showing the distribution of CHIP driver genes across the TOPMed cohort (4938 CHIP mutations in 4229 individuals). More than 75% of mutations were in either DNMT3A, TET2, or ASXL1. [file mmc7.pdf]

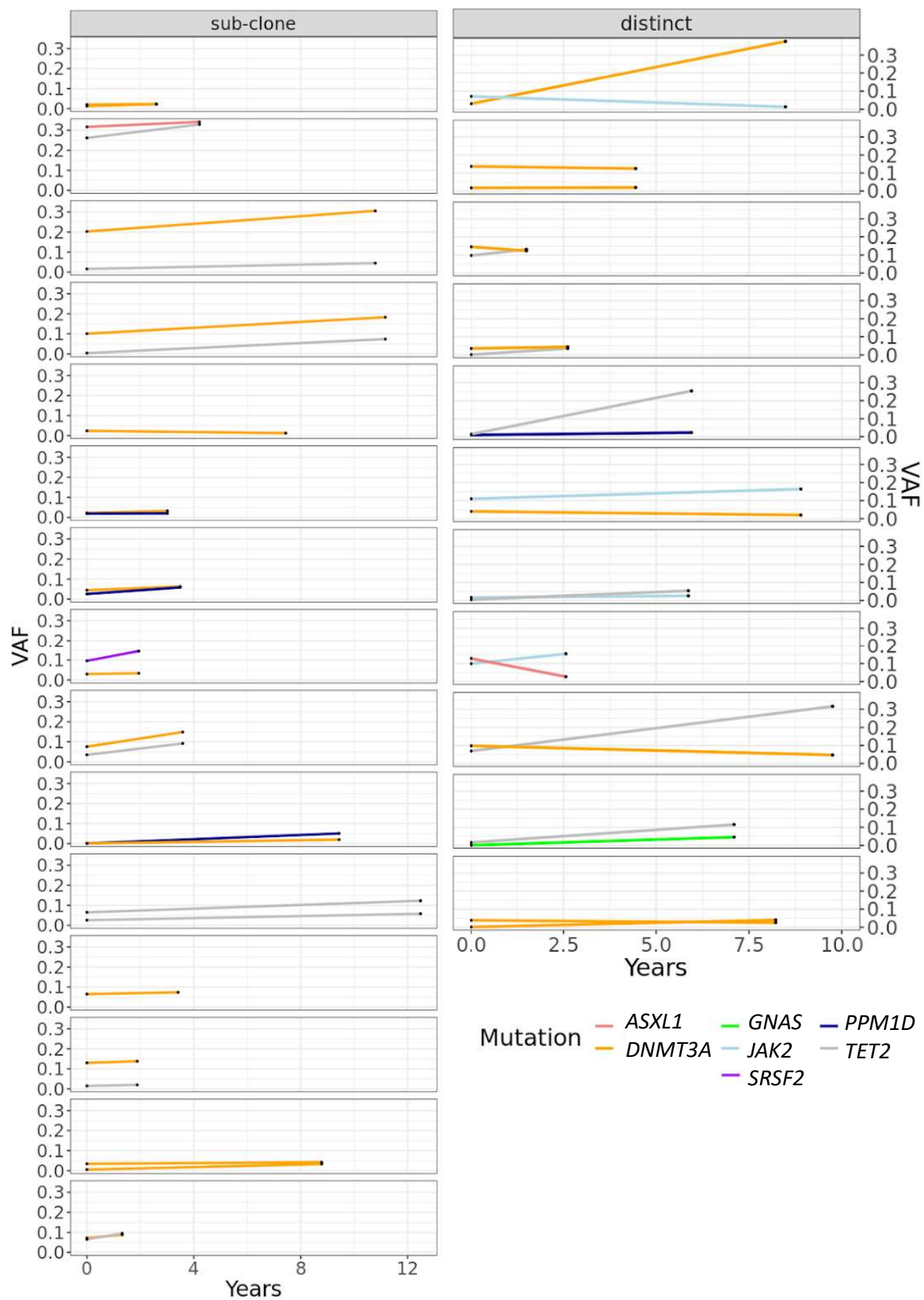

Supplement: Supplemental Figure S3 — Individual clonal dynamics over time. Plot showing the clonal behavior in each individual with two CHIP driver mutations. Each line represents one clone, and each small plot represents an individual from the cohort. Facets on the left are categorized as sub-clonal, while facets on the right are categorized as distinct clones. [file mmc8.pdf]

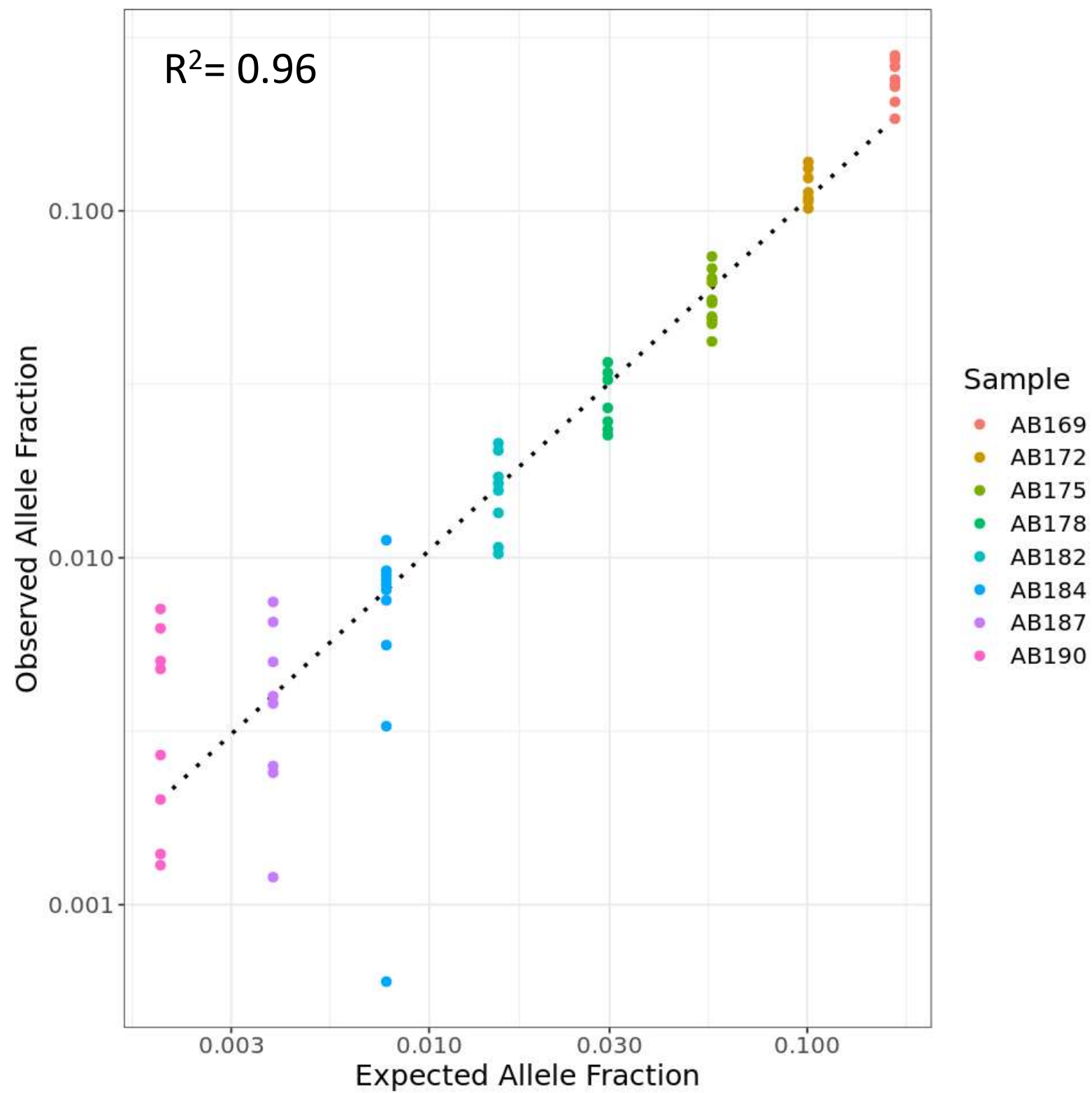

Supplement: Supplemental Figure S4 — Limit of detection test for CHIP detection assay. Results of a limiting dilution experiment in which a DNA sample with known genotype was combined at serial fixed ratios with a second sample of known genotype. Each color represents a distinct sample, and the correlation line is displayed to show the correlation between observed and expected allele fraction. This method robustly detects variants present in >1% of DNA. Beneath this 1% threshold, variants down to approximately 0.1% allele fraction are detected, but with less accuracy for the estimated allele fraction. [file mmc9.pdf]
